# A Design of Irregular Grid Map for Large-Scale Wi-Fi LAN Fingerprint Positioning Systems

**DOI:** 10.1155/2014/203419

**Published:** 2014-09-15

**Authors:** Jae-Hoon Kim, Kyoung Sik Min, Woon-Young Yeo

**Affiliations:** ^1^Department of Industrial Engineering, Ajou University, 206 Worldcup-ro, Yeongtong-gu, Suwon 443-749, Republic of Korea; ^2^International Cooperation Group, Korea Internet & Security Agency, 109 Jungdae-ro, Songpa-gu, Seoul 138-803, Republic of Korea; ^3^Department of Information and Communications Engineering, Sejong University, 98 Gunja-dong, Gwangjin-gu, Seoul 143-747, Republic of Korea

## Abstract

The rapid growth of mobile communication and the proliferation of smartphones have drawn significant attention to location-based services (LBSs). One of the most important factors in the vitalization of LBSs is the accurate position estimation of a mobile device. The Wi-Fi positioning system (WPS) is a new positioning method that measures received signal strength indication (RSSI) data from all Wi-Fi access points (APs) and stores them in a large database as a form of radio fingerprint map. Because of the millions of APs in urban areas, radio fingerprints are seriously contaminated and confused. Moreover, the algorithmic advances for positioning face computational limitation. Therefore, we present a novel irregular grid structure and data analytics for efficient fingerprint map management. The usefulness of the proposed methodology is presented using the actual radio fingerprint measurements taken throughout Seoul, Korea.

## 1. Introduction

**Figure 1:**
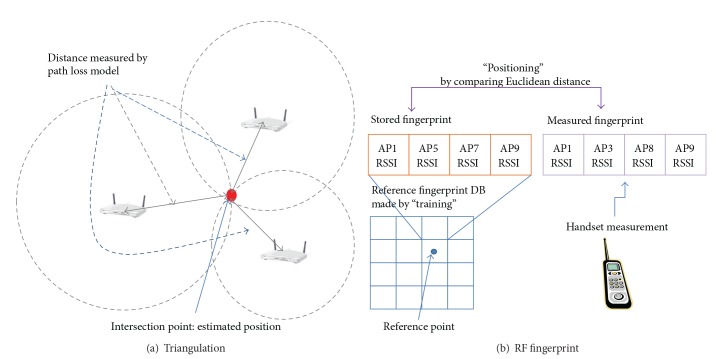
Position estimation by WPS.

A location-based service (LBS) coordinates user location with various end-user applications to improve relevance, context, and economic value. Despite the many possibilities offered by an LBS, its market penetration has been slow. Most early-stage services have failed to proliferate a mass market. Moreover, monetization of the services is limited to a few special-purpose markets, such as car map/navigation. The limitations of LBSs are related mainly to the insufficient precision of position estimation. The general mean error of position estimation is in the order of many tens of meters and the deviation can be in the order of hundreds of meters. With the rapid increase in Wi-Fi LAN access points (APs) in metropolitan areas, Wi-Fi can be used as a viable alternative positioning infrastructure [1, 2]. Each Wi-Fi AP continuously generates a radio signal with a unique identifier or media access control (MAC) address, which enables mobile devices to identify a specific AP. The millions of public and private Wi-Fi APs can be used for Wi-Fi-based positioning. Based on the Received Signal Strength Index (RSSI) from each valid AP and embedded algorithms, the typical accuracy of Wi-Fi positioning is in the order of tens of meters in metropolitan areas, which is more accurate than other cellular positioning technologies, because Wi-Fi APs are more closely spaced than cellular network base stations. The TTFF can be as short as 100 ms. Compared with a GPS [3, 4], Wi-Fi positioning works better in urban canyons than in rural areas. It works well in dense metropolitan areas, both outdoors and indoors, owing to its greater received signal strength and lower attenuation. In many Wi-Fi positioning methods, AP triangulation and radio frequency (RF) fingerprinting provide the basic scientific significance to current positioning methodologies. Triangulation is simple to implement [1, 5, 6]. As seen in Figure 1(a), three reference APs with already known coordinates are required. After measuring the distance from the APs and a target point, three circles can be drawn. The circles intersect at a single target point. The coordinate of the target point can be easily calculated by the distance from the known coordinates of the APs.

The significant difficulty of this approach is the distance measurement from each AP to the target point. Typical path loss models such as COST231 [7] and Okumura-Hata [8] are generally applied to measure the distance. However, it is extremely difficult to build a good, general model for distance measurement, which coincides with the actual field situation. RF fingerprinting [9, 10] consists of two phases—training and positioning—demonstrated in Figure 1(b). In the training phase, a reference fingerprint database (DB) is constructed. The reference DB contains the signal strength measurements of the APs at all reference points. Usually, the entire area should be divided into a set of grids and the centers of grids are considered as the reference points. During the positioning phase, the position of a target point can be identified by comparing its measured fingerprint with the prestored reference fingerprints DB. The main advantage of RF fingerprinting is algorithmic simplicity. Simple comparing algorithms, such as pattern matching, can be easily applied to a practical process of position estimation. Then the RF fingerprinting is more preferred than triangulation [11].

Most of advancements for RF fingerprinting have been searched in position estimation algorithms. The most well-known pattern matching algorithm is nearest neighbor (NN) [9]. As an enhanced version of NN algorithm, K nearest neighbor (KNN) algorithm can be taken into account [9]. The average of coordinates of k-reference grids can be used to determine the estimated position of a target point. Various variations, such as smallest polygon [12] and neural networks [13], are applied in the framework of KNN pattern matching. Another type of algorithm for positioning adopts a probabilistic framework. The idea of the probabilistic framework is to compute the conditional probabilistic density function (pdf) of an estimated position of the target point. The probabilistic likelihood can be modeled by histogram [14], Gaussian [13], Lognormal [15], or Kernel [14]. In addition, a hybrid method of pattern matching and probabilistic frame was invented [16]. Using the overlapped probabilistic existence maps of APs, they calculated the most promising position of a target point. Another significant study for RF fingerprinting is data filtering [17]. To keep the integrity of fingerprint data, data filtering schemes, such as the Kalman-filter [18] or probabilistic filtering approach with machine learning [14], are applied to Wi-Fi positioning for fingerprint data management.

In spite of such advancement of position estimation algorithms or data filtering methods, the enhancement of positioning precision currently faces computational limitation. The error bound still remains to tens of meter. The recent algorithmic advances just present small and limited improvement. Therefore, we focus on the more fundamental frame of RF fingerprint WPS: the structure of reference fingerprint DB. The conventional large-scale fingerprint DBs have a regular square map structure. The entire area of a geographical region is divided into many regular square grids [10]. The measured fingerprints are allocated to each grid. If multiple fingerprints are measured in a single grid, the multiple fingerprints are merged into a single fingerprint. Shin and Cha [19] construct a topological map with regular Wi-Fi signal calibration points, assign semantically meaningful labels into the map, and estimate the semantic location of the user based on the current Wi-Fi observation. Chan et al. [20] and Kim et al. [21] build autonomous and collaborative RF fingerprint localization systems. They both use an indoor regular grid formation by anonymous mobile users who automatically collect data in daily life without purposefully surveying an entire building. Brunato and Battiti [22] use many regression-based algorithms to estimate position over the regular grids in indoor environment. Nafarieh and Ilow [23] build a testbed with off-the-shelf equipment and the corresponding applications over the regular grid formation. The generality of a regular grid formation is also shown in metropolitan area applications. The study of Taipei [24] contains city-scale data samples. They use uniform calibration points in the city. In the work of Seattle [25], they collect a wide-scale training trace for the entire Seattle area. The trace data are stored in a radio map which has regular trace routes and a grid formation.

**Figure 2:**
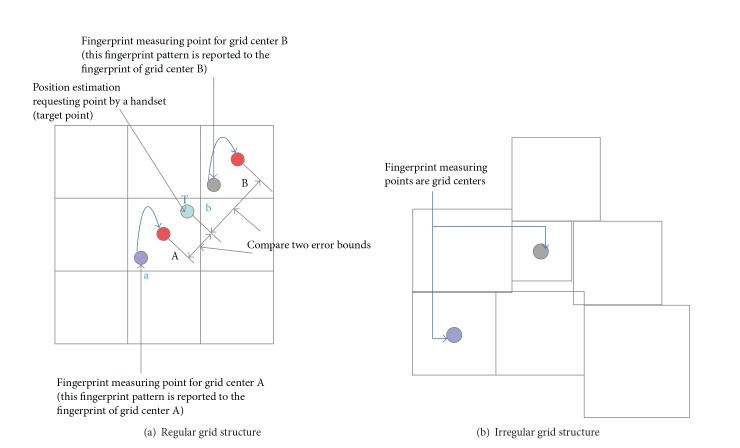
Comparison of regular and irregular forms of grids.

The regular grid fingerprint DB has a critical drawback to estimate precise position. In a general RF fingerprint WPS, the estimated position of a target point, that is, the position estimation point requested by a handset, is usually calculated by the weighted average of the fingerprint positions with the estimated probability. The regular grid structure merges the collected fingerprints to fit the regular grid segmentation, and the merged fingerprint positions are arranged to the center points of the grids. Then, the multiple candidate grids are selected by a position estimation algorithm. The center points of candidate grids are used to calculate the estimated position of a target point. That is, the center points of grids have a significant role in the position estimation.  Figure 2(a) shows the misleadingness for center points in a regular grid fingerprint DB. In the case of a regular grid structure, the center point is assumed to be a representative point of merged fingerprints. Thus, the target point is easily misestimated. The target point *T* is definitely geographically close to the point *A*, but the measured fingerprint by a handset in *T* is more similar to the fingerprint of *b* than the fingerprint of *a*. Here, the fingerprint of *a* is allocated to the center point *A* and the fingerprint of *b* is allocated to the center point *B*. Then, the grid, which has center point *B*, is selected as one of candidate grids for a position estimation algorithm. The irregular grid setup in Figure 2(b) eliminates the aforementioned incorrect grid selection. Each measured fingerprint is fully analyzed: some of the fingerprints can be clustered and integrated under the statistical significance test. Then, the finally validated fingerprint by the significance test has its statistical significance among the other fingerprints in a reference DB.

The area of a grid in an irregular grid structure presents additional important information for position estimation. Each grid shows the dominant coverage of the specific fingerprint that has distinct statistical significance. The dominant coverage directly means the error bound of position estimation. The usual RF fingerprinting estimates the position of a target point as a center of grid. But, the actual position of the target point can be located anywhere in the selected grid. The coverage of fingerprint (i.e., grid) gives important information for error limit.

In this paper, we propose a design of irregular grid structure for RF fingerprinting and a statistical significance test framework for fingerprint data validation. The sufficiently valid fingerprint data are established by the proposed significance test and the grid area of each fingerprint is maintained with an effective level with an irregular form. As a result of the proposed significance test framework, we can create a standard RF fingerprint DB that consists of an effective, valid dataset. All fingerprint data used in the developed testbed are harvested from actual radio fingerprint measurements taken throughout Seoul, Korea. This demonstrates the practical usefulness of the proposed framework.

## 2. Irregular Grid Segmentation of Fingerprint DB

**Figure 3:**
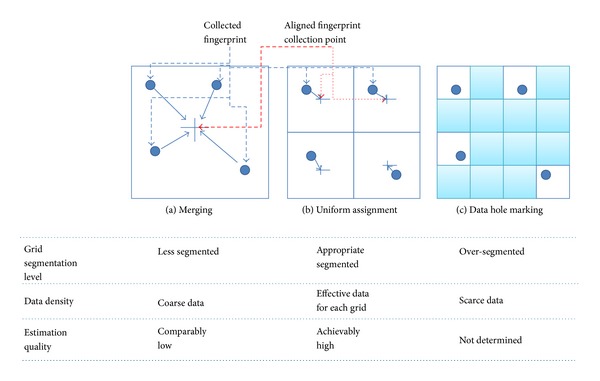
Grid segmentation level and fingerprint data alignment.

Grid size is one of the main concerns of RF fingerprint WPS. Almost all the fingerprint data are collected by automatic scanning (usually using a vehicle) [26]. Then, the fingerprint data should be aligned to each grid in a reference fingerprint DB.  Figure 3 shows three representative fingerprint alignment methods.

**Figure 4:**
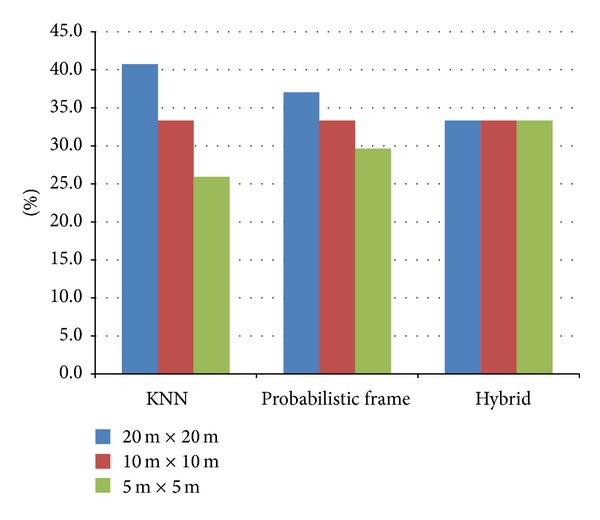
Performance comparison for grid segmentations.

The most desirable situation is the uniform assignment case. Fingerprint collection in the training phase is uniformly performed on a grid map. A single, effective fingerprint can be assigned to each grid. Then, the estimation quality can be guaranteed. However, in most cases, fingerprint collection cannot be performed uniformly. The collected fingerprints are scattered: partially dense and partially scarce. Moreover, fingerprints are probably not collected at the center of grids because it is not easy to collect fingerprint data from the exact center of a regular grid. Even for the uniform assignment illustrated in Figure 3, the difference between aligned and actual collection point produces additional alignment error.  Figure 4 shows the performance for three types of conventional single-size regular segmentation: 20 m × 20 m, 10 m × 10 m, and 5 m × 5 m. To test grid segment variations, we organized three types of grid maps from the same raw fingerprint data. The collected raw fingerprint data can be simply merged into a single grid. Further, small-size grid segmentation can contain many fingerprint data holes. To compare the performance among the grid segmentations, we select 27 target points for position estimation and then we applied three representative estimation algorithms: KNN [9], probabilistic frame [14], and a hybrid method of pattern matching with probabilistic frame [16]. The bar chart in Figure 4 presents the ratio of grid segmentation which has the minimum estimation error (i.e., in case of KNN, 40.7% of target points have the best estimation quality in 20 m × 20 m segmentation, 33.3% in 10 m × 10 m, and 25.9% in 5 m × 5 m).

Figure 4 strongly intends the performance indifference among regular grid segmentations. It is hard to find any relation between segmentation size and position estimation quality. This indifference comes from the ineffectiveness of a single-size regular grid segmentation and alignment method (i.e., simple merging and data hole marking). The simple merging or data hole marking for regular grid segmentations does not give any sufficient compensation effect on the collected fingerprint data. Therefore, we propose a totally novel structure for fingerprint DB map: variable and irregular grid segmentation.

**Figure 5:**
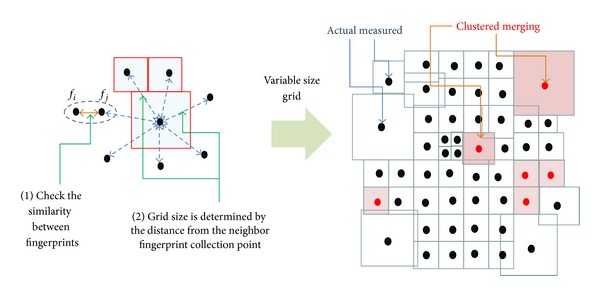
Making irregular grid segmentation.

Figure 5 shows the irregular grid segmentation by clustered merging. We replace simple merging with the clustered merging by the significance test, which ensures effective alignment of collected fingerprints to their respective grids. Fingerprints collected using any scanning devices can be appropriately operated (i.e., similarity test described in Section 3) and assigned to flexible-size grids. The grid size of a fingerprint is determined by the geographical relationship with the neighbor fingerprints. We can find the average distance to the neighbor fingerprint collection points and assign the proper grid size to each fingerprint. The valid fingerprint data stored in irregular grids guarantee the efficiency of data management and enhanced accuracy of position estimation in RF fingerprint WPSs. Additionally, the flexibility of the grid segmentation can be a useful strategy for the fingerprint collection process: a dense segmentation in commercial areas and a light segmentation in residential or rural areas. Next, we build the clustered merging by significance test framework to establish valid fingerprint data.

## 3. Clustered Merging by Significance Test

The usual fingerprint collection devices are operated automatically: they measure fingerprint along the collection routes and store the measured fingerprints to a reference fingerprint DB without data calibration. Because of the automatic collection process, we can find lots of close fingerprint groups. The fingerprints in each group are collected at very close points with very similar fingerprint patterns. These fingerprints in a close group are hard to be assigned to the separated grids. Any position estimation algorithms have their own inherent error bound. Each of the small-size grids, within the inherent error bound, does not have its statistical differentiation. The clustered merging for the close fingerprint groups can generate statistically distinct fingerprints. Each fingerprint generated by the proposed clustered merging has sufficient statistical validity to each grid. The study of Kaufman and Rousseeuw [27] demonstrated the recent researches for group clustering. In an ordinary clustering, the member of cluster has a scalar value or a simple vector form (the elements of vector have solid and deterministic values). An RF fingerprint has also a vector form shown in Figure 1. But all elements of a fingerprint vector are random variables. An RF fingerprint, a vector of random variables, has mathematical difficulty to be applied to an ordinary clustering. Therefore, we develop a special statistical tool for fingerprint clustering: significance test on fingerprints.

The proposed significance test is performed using the geographically close fingerprint group. The statistical difference between two fingerprints is based on the square of the Euclidean distance (*d* ^2^(*i*, *j*)) between the two fingerprint pairs (*f*_*i*_,*f*_*j*_). The Euclidean distance is a very well-known metric to measure the difference between two vectors. Feng et al. [28] adopted the Euclidean distance to measure the difference (or similarity) between two fingerprints with vector form as follows:

(1) $$\begin{array}{c} d^{2}\left(i,j\right)={\left(f_{i}-f_{j}\right)}^{2}, \end{array}$$

where *f*_*i*_ = {RSSI_AP_1__^*i*^, RSSI_AP_2__^*i*^,…, RSSI_AP_*n*__^*i*^}, *f*_*j*_ = {RSSI_AP_1__^*j*^, RSSI_AP_2__^*j*^,…, RSSI_AP_*n*__^*j*^}.

**Figure 6:**
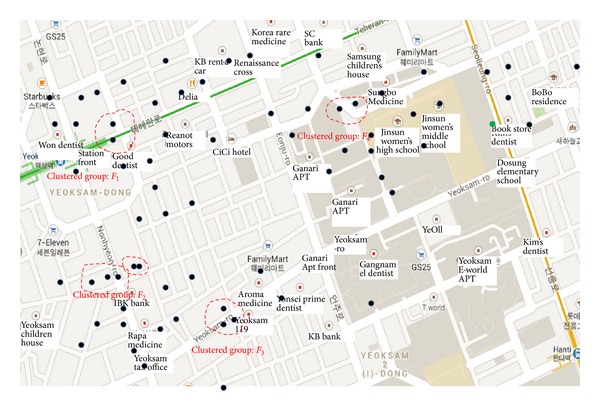
Clustered groups for the significance test.

Each value of RSSI_AP_*k*__^*i*^ (RSSI for AP_*k*_ in fingerprint *f*_*i*_) is a random variable and has a measurement error that tends to follow a normal distribution. Thus, each element of vector *f*_*i*_ − *f*_*j*_ also follows a normal distribution. By transforming the elements of vector *f*_*i*_ − *f*_*j*_ to the standard normal distribution, *d* ^2^(*i*, *j*) tends to follow a chi-square distribution with a degree of freedom *n*; that is, *d* ^2^(*i*, *j*) ~ *χ* ^2^(*n*). Generally, the *χ* ^2^(*n*) has a mean *n* and variance 2*n*. Moreover, the $\sqrt{2d^{2}(i,j)}$ is approximately normally distributed with a mean $\sqrt{2n-1}$ and unit variance [29]. Based on the aforementioned statistical characteristic, we can determine a difference between the two fingerprints (*f*_*i*_,*f*_*j*_). When the calculated $\sqrt{2d^{2}(i,j)}$ value is greater than a certain threshold, *f*_*i*_ and *f*_*j*_ are determined to be two statistically different fingerprints (the calculation of a practical threshold is described in Section 4). However, this significance test between only two fingerprints has limitations when it is applied to the practical clustered merging. The significance test should be applied on a fingerprint group basis in an entire area. (Example groups are illustrated in Figure 6.)

Figure 6 shows examples of clustered groups (*F*_1_ ~ *F*_4_). Each clustered group has geographical proximity. The fingerprint members in the same clustered group are determined by the following inclusion test:

(2) $$\begin{array}{c} \text{Inclusion test: }\sqrt{2d^{2}\left(f_{i},F_{l}\setminus f_{i}\right)}\leq \text{inclusion}\;\text{threshold}, \end{array}$$

where *d* ^2^(*f*_*i*_, *F*_*l*_∖*f*_*i*_) = square of Euclidian distance between *f*_*i*_ and mean(*F*_*l*_∖*f*_*i*_) and mean(*F*_*l*_∖*f*_*i*_) denotes an artificial fingerprint with elements that are the mean values of elements for all fingerprints in *F*_*l*_, excluding *f*_*i*_. A fingerprint *f*_*i*_ is set to be a member of the clustered group *F*_*l*_ when $\sqrt{2d^{2}\left(f_{i},F_{l}\setminus f_{i}\right)}$ is less than an inclusion threshold. An inclusion threshold is applicable as same as aforementioned two fingerprints cases. Some fingerprints can be considered to be clustered with multiple groups (see the clustered group *F*_2_ and its proximity in Figure 6). We perform a separation test for discriminating the clustered group partitions. Consider

(3) $$\begin{array}{c} \text{Separation test: compare}\;d^{2}\left(f_{i},F_{l1}\right)\;\text{and}\;d^{2}\left(f_{i},F_{l2}\right). \end{array}$$

If *f*_*i*_ is a candidate member of both clustered groups *F*_*l*1_ and *F*_*l*2_, we compare a square of Euclidian distance between *f*_*i*_ and mean(*F*_*l*1_) (also, mean(*F*_*l*2_)). *f*_*i*_ is added to a closer group in view of the Euclidian distance.

The aforementioned inclusion and separation test guarantee the strict group partition for clustered merging. However, as there are many fingerprints to be tested, we develop a practical clustered merging procedure that has an order of tested fingerprints. See Algorithm 1.

At the beginning of the procedure, all fingerprints belong to *unmarked* *set* *S*. A fingerprint *f*_*i*_ is selected in the set *S*. Then, the significance test is performed between the selected fingerprint and one of its neighbor fingerprints (*j* ∈ *neighbor*(*i*)). Note that the selected fingerprint and its neighbor fingerprints share geographical proximity.

## 4. Numerical Results

**Figure 7:**
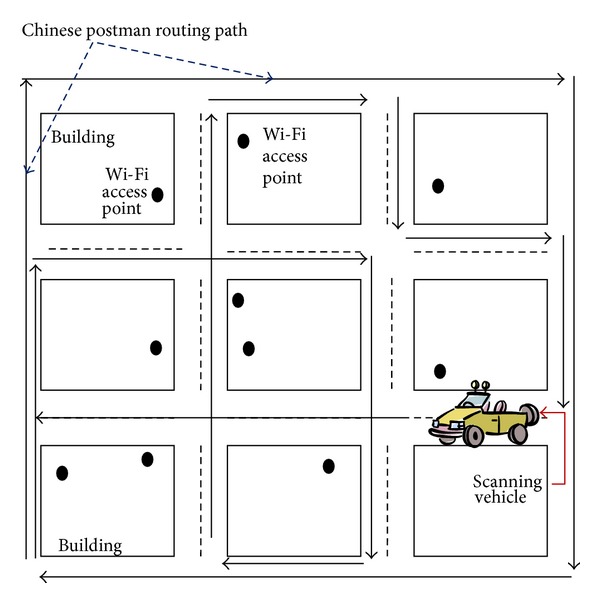
Scanning of RF fingerprints.

To demonstrate the applicability of the proposed method, we collected whole fingerprint data from the Seoul Gangnam urban district. The WPS practices in actual city were performed in some previous works. In work of Cheng et al. [30], the 30-minute restricted scanning tried to approve the practicability of WPS. The work of Yoshida et al. [26] shows a 100 m × 100 m test district with 873 APs. The study in Sydney [10] has a 500 m × 800 m test district with 1300 APs and 172 reference grids. We significantly extended the applicability of fingerprint WPS to a metropolitan area of Seoul (the area of Seoul Gangnam is 39.55 km^2^) [31]. A single scanning process for Gangnam district usually generates 0.6 million fingerprint data. The scanning process was performed three times to collect fingerprint data. The total volume of collected fingerprint data exceeded 1.8 million fingerprints. This is a huge amount of data in a relatively large area.  Figure 7 shows a simplified diagram for fingerprint collection by a scanning vehicle. A scanning vehicle runs through the metropolitan area to make an entire RF fingerprint DB in a form of a geographical map. For efficient fingerprint collection, a fingerprint collector segments an entire area into multiple fractions and builds efficient scanning routes for each fraction. The most popular method of building scanning routes is Chinese Postman Routing. Chinese Postman Routing is a very well-known postman tour or route inspection method of finding the shortest closed path or circuit that visits every edge of an (connected) undirected graph. This method can be used to obtain the optimal Eulerian circuit (a closed walk that covers every edge once).

The scanning vehicle includes a diagnostic machine (DM) with GPS receiver. The collected fingerprint data are stored in a temporary storage in a notebook PC connected to a DM. After the single collection route, the whole fingerprint data are transferred to a central storage for reference DB map. The usual Wi-Fi fingerprints are collected in the form of Table 1.

A Wi-Fi fingerprint consists of the following: a base station identification (BSSID; i.e., MAC address), service set identification (SSID), measurement *x*-axis (MES_*X*, i.e., longitude), measurement *y*-axis (MES_*Y*, i.e., latitude), and Received Signal Strength Index (RSSI). When an AP is detected by an automatic scanning device, the fingerprint data, that is, BSSID, SSID, and RSSI, are stored with their position, that is, MES_*X* and MES_*Y*.

**Figure 8:**
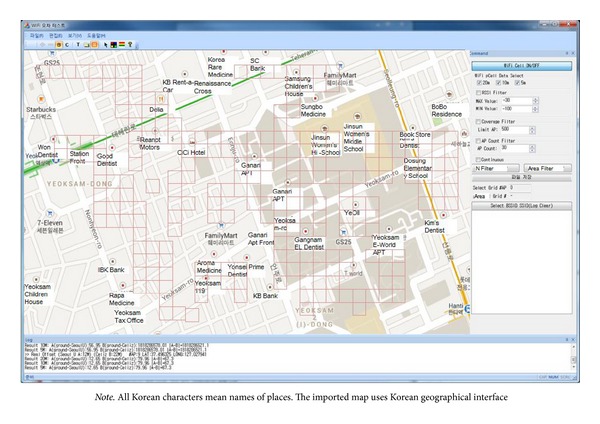
Example of single-size regular grid segmentation.

As the first step, we applied the proposed data alignment method and evaluated its performance in a relatively restricted area for visualization (see the window-based performance analysis tool as shown in Figure 8). The test area is a square district (550 m × 350 m) in Seoul Gangnam. This district is classified as a commercial area that includes many commercial buildings and dense foot traffic. In total, 1,682 APs were detected and 459 fingerprint data were measured. Figure 8 contains a single-size regular grid (20 m × 20 m) segmentation. There were 241 grids of regular size, and 52.3% of the sample area was covered by single-size grids.

**Figure 9:**
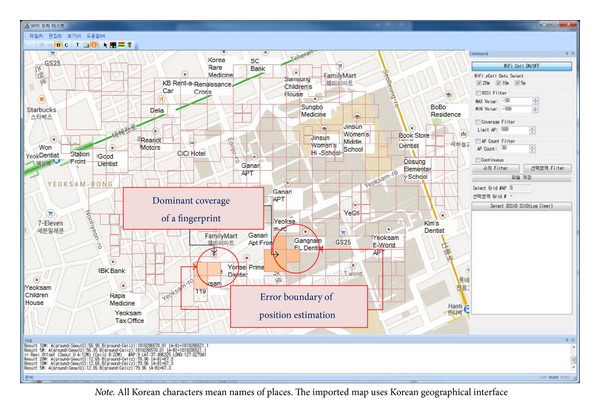
Example of irregular grid segmentation.

Next, we applied irregular grid segmentation by clustered merging.  Figure 9 shows the new segmentation of the sample area shown in Figure 8.

**Figure 10:**
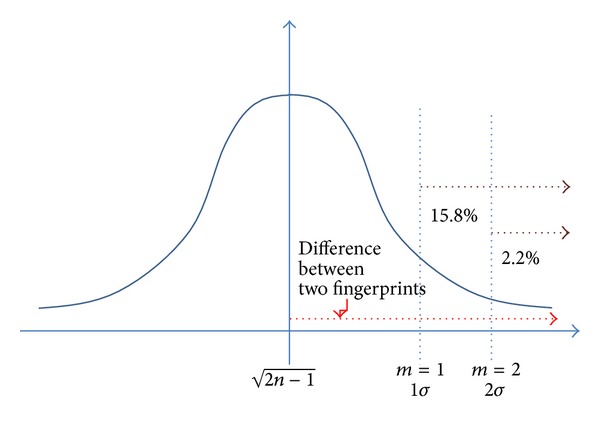
Statistical threshold specification.

The proposed fingerprint data alignment method reconstructs entire grids in the sample area. In total, 289 grids were generated with irregular size. The total area covered by the proposed method was 72.1% of the sample area. The threshold value, which was applied to the significance and inclusion tests, was determined by the statistical characteristic of the fingerprint difference. The fingerprint difference, that is, $\sqrt{2d^{2}(i,j)}$ in Section 3, tends to follow a normal distribution with mean $\sqrt{2n-1}$ and unit variance; that is, *σ* = 1. Ideally, we can determine the two fingerprints, whose difference (i.e., $\sqrt{2d^{2}(i,j)}$) is greater than zero, as two separated vectors. However, all elements and their mathematical variations are random variables because of their measurement imperfectness. That is, the mean $\sqrt{2n-1}$ is observed as an average difference between two fingerprints exactly speaking, $\sqrt{2}\times (\text{Euclidean}\;\text{distance}\;\text{of}\;\text{two}\;\text{fingerprints})$. We can specify a certain relative difference level from an average difference to determine similarity between two fingerprints. For example, when an average difference is observed as a value *a*, we can set *ka* (0 < *k* < 1) as a threshold for significance test. Now, finding a normal distribution of $\sqrt{2d^{2}(i,j)}$, we can use $\sqrt{2n-1}-m$ as a useful threshold value; that is, relative difference level can be controlled by *m*. By the simple specification of *m*, we can make a statistically meaningful threshold. If *m* = 1, then the two fingerprints, which have a difference belonging to a lower 15.8% of the whole difference distribution, are determined to be statistically different. To test the effectiveness of irregular grid segmentation, we set *m* = 2, that is, lower 2.2% of the total difference distribution, and compared the regular grid segmentation (see Figure 10).

**Figure 11:**
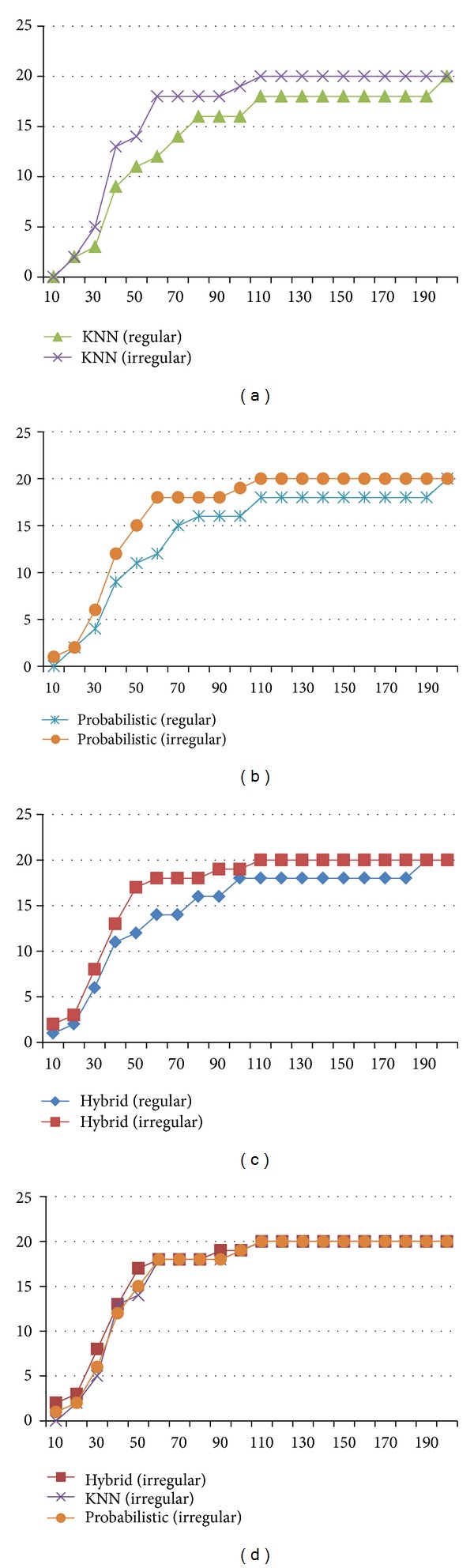
Position estimation error for sample points.

Figure 11 shows detailed results of the position estimation for 20 sample target points. The *x*-axis denotes the estimation error in meter and the *y*-axis denotes the cumulative number of sample target points. The irregular grid segmentation generated superior estimations for all the target points for all the applied estimation algorithms (e.g., for KNN, 18 points have the error bound within 55 m in case of irregular segmentation, but only 12 points within 55 m error bound in case of regular segmentation). Three representative estimation algorithms were applied: KNN [9], probabilistic frame [14], and a hybrid method of pattern matching with probabilistic frame [16].  Figure 11(d) shows the performance difference among estimation algorithms. The hybrid method has slightly higher performance compared to KNN or probabilistic method.

Honestly, a part of contribution is due to the increment of grids. 19.9% increased grids of irregular grid segmentation (the difference between regular and irregular grids is 48) make more precise position estimation. However, we could observe much higher extra enhancement. Even considering the 19.9% increment of grids, the 37.5% precision enhancement is observed for hybrid positioning method, 34.7% for KNN, and 35.1% for probabilistic.

**Figure 12:**
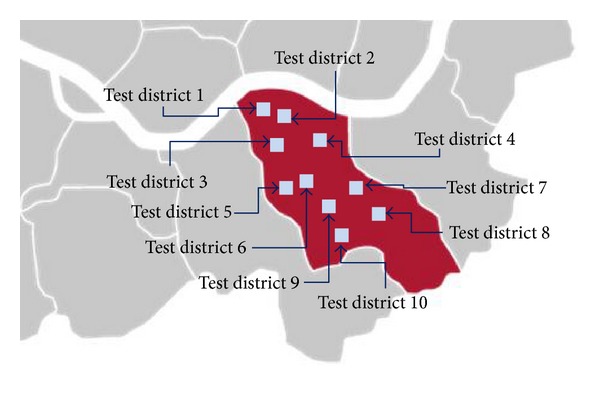
Test districts in Seoul Gangnam.

Next, we extended our irregular grid segmentation using the significance test in a large area. Ten test districts (see Figure 12) in Seoul Gangnam were selected to prove the applicability of the proposed irregular grid segmentation. The area of Gangnam district is 39.55 km^2^. The range of area for test districts is 0.10 km^2^ ~ 0.17 km^2^. Each district has 50 ~ 60 target points for position estimation.

**Figure 13:**
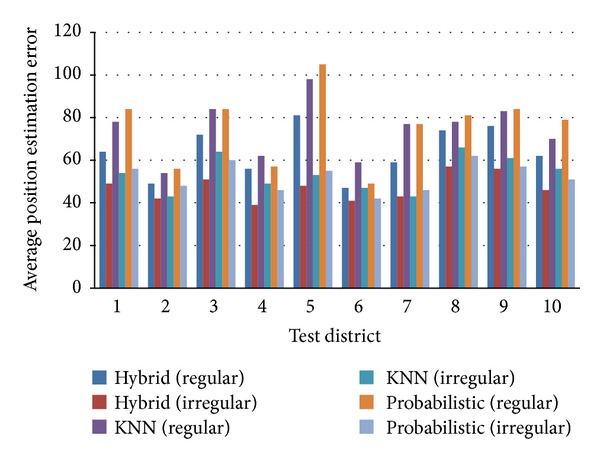
Comparison of regular and irregular grid segmentation.

Figure 13 shows the estimation results which prove the effectiveness of the proposed method in various diversified environments of an urban area. For comparison purposes, Figure 13 contains the estimation error of the regular grid segmentation for three representative estimation algorithms (KNN, probabilistic, and hybrid). We measured on average 30% enhancement for KNN, 29% for probabilistic, and 26% for hybrid position estimations.

To show the effectiveness of the proposed irregular segmentation itself, the numbers of grids for 10 districts are presented in Table 2.

The average increment of grids was 11.2%. However, 26.9% precision enhancement was observed for hybrid positioning method, 25.7% for KNN, and 30.1% for probabilistic. The presented comparison between “increment of grids” and “precision enhancement” shows the significant advantage of irregular grid segmentation. The simple increment of grids cannot guarantee such a significant enhancement.

**Figure 14:**
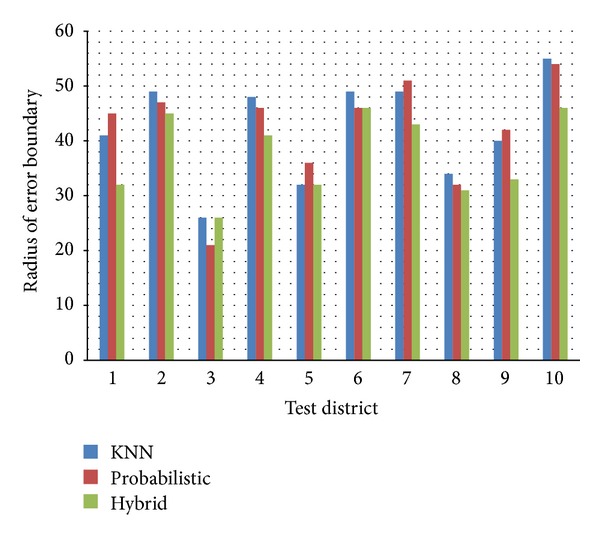
Comparison of radius of error boundary.

Additionally, we were able to show the radius of the error boundary (see Figure 14). The position estimation algorithm deployed in the experiment selected the best matching grids in multiple candidate grids, as shown in Figure 9. The circular boundary of the candidate grids was considered as the estimation error boundary. The proposed irregular grid segmentation was better able to illustrate the dominant coverage of each fingerprint. Then, the radius of error boundary can be useful for the qualification of position estimation.

Note that the majority of test points belong to the outdoor environment. The automatic scanning vehicle has a problem to access indoor environment. Most of the indoor fingerprints are collected by human power. Thus, our experiment has a limitation for the applicability on the indoor environment. However, our proposed segmentation is applicable both on indoor and on outdoor environment. The radio signal fluctuation and structure complexity are more serious in the indoor environment. The proposed irregular segmentation has relative advantage on the complex and fluctuated environment. We carefully expect the effective applying to the indoor environment.

## 5. Conclusion

The rapid growth of mobile communication and the proliferation of smartphones have drawn significant attention to location-based services. One of the most important factors in the vitalization of LBS is the accurate position estimation of a mobile device. Traditional triangulation has an inevitable weakness when estimating the exact position of an AP. Moreover, significant technical advances are not shared publicly by solution providers. An RF fingerprint WPS is another valuable way to penetrate the positioning solution provider market. Even using indiscriminate fingerprint collections, providers can build an approximate fingerprint DB and apply a simple pattern-matching algorithm for position estimation. However, to build a competitive fingerprint WPS solution, we should focus on the fingerprint data management and precise estimation algorithms. Much of enhancement on fingerprint WPS focuses on an estimation algorithm itself. However, the improvement by estimation algorithms faces limitation. The more essential factor of fingerprint WPS is a structure of radio fingerprint map. Because of the geographical complexity of urban areas, similar, even duplicated, fingerprint data are collected at close points. Therefore, we presented a data clustering method and irregular grid segmentation. Based on the statistical significance test for fingerprints, collected fingerprints were merged. Each fingerprint grid had an irregular form to cover the geographical area. The proposed new fingerprint data management for position estimation can strengthen the advantages of the fingerprint WPS. Compared with conventional fingerprint data alignment approaches, our method achieves better performance in both average error of estimation and deviation of errors. Furthermore, all the fingerprint data were harvested from an actual measurement of RF fingerprints from the Gangnam district, Seoul. We built an irregular fingerprint map for an entire area of Seoul and applied position estimation. These trials show the practical usefulness of the proposed methodology.

## Figures and Tables

**Algorithm 1:**
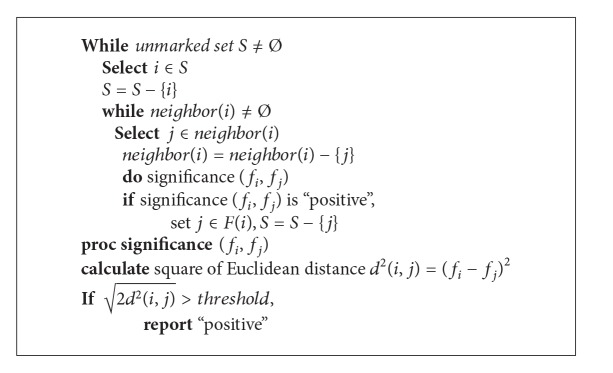


**Table 1:** Example of a Wi-Fi AP fingerprint.

BSSID	SSID	MES_*X*	MES_*Y*	RSSI (dB)
00:01:36:1f:9c:d2	sklifeap_4	37.5036	127.0336	−80
00:01:36:24:66:60	primebc-ap2	37.5060	127.0436	−69
00:01:36:25:2a:ea	Hpsetup	37.4956	127.0302	−79
00:01:36:25:2a:eb	SK_WLAN	37.4956	127.0302	−79
00:01:36:26:24:27	KWI-B2200T-	37.5038	127.0274	−87
00:01:36:26:24:28	D-1201	37.5038	127.0274	−83
00:01:36:27:4d:54	Tectura Corporation	37.5084	127.0434	−89
00:01:36:2a:84:f9	Default	37.4962	127.0302	−81

**Table 2:** Grids difference for 10 districts.

District	1	2	3	4	5	6	7	8	9	10
Regular	256	312	261	292	304	297	214	223	196	269
Irregular	287	332	296	338	341	332	248	236	221	286
